# Risk Factors of Keratoconus in High School Students in Trinidad and Tobago: A Cross‐Sectional Study

**DOI:** 10.1002/hsr2.71980

**Published:** 2026-04-11

**Authors:** Ngozika Esther Ezinne, Michael Agyemang Kwarteng, Shinead Phagoo, Ameera Roopnarinesingh, Khathutshelo Percy Mashige, Uchechukwu Levi Osuagwu

**Affiliations:** ^1^ Optometry Unit, Department of Clinical Surgical Sciences University of the West Indies, Saint Augustine Campus Saint Augustine Trinidad and Tobago; ^2^ Bathurst Rural Clinical School Western Sydney University Bathurst Australia; ^3^ Translational Research Health Institute Western Sydney University Campbelltown Australia; ^4^ Discipline of Optometry University of KwaZulu‐Natal Durban South Africa

**Keywords:** atopy, family history, high school students, keratoconus, risk factors, sunlight exposure, Trinidad and Tobago

## Abstract

**Background:**

Keratoconus (KC) is the most common form of keratectasia that is associated with impaired vision and reduced quality of life, especially in adolescents. This study aims to identify and assess the risk factors associated with KC among high school students in Trinidad and Tobago (T&T).

**Method:**

This was an institutional‐based, cross‐sectional study that utilized the Keratoconus (KC) Risk Investigative (KRIS) questionnaire to assess risk factors of KC among high school students in T&T. A multistage stratified cluster sampling technique was employed. The study examined established and non‐established risk factors.

**Results:**

A total of 2084 students (mean age: 14 years, SD = 1.6, range 11–20) participated, with 674 (32.3%) reporting at least one risk factor of KC. The most prevalent risk factor overall was prolonged near‐work (≥ 8 h/day, 59.8%), while exposure to sunlight (25%) was identified as the most established risk factor. Of those with risk factors, 60 (2.9%) had KC which was significantly associated with family history (*χ* ^2^ = 13.30, df = 1, *p* < 0.001) of KC, allergic diseases (*χ* ^2^ = 5.54, df = 1, *p* = 0.019), and light sensitivity (*χ* ^2^ = 10.18, df = 1, *p* = 0.001). Adjusted logistic regression showed that those with a family history of KC were 2.56 times more likely to develop the condition (95% CI: 1.11–5.93, *p* = 0.028), and those with light sensitivity had nearly double the odds (OR = 2.10, *p* = 0.007).

**Conclusion:**

This study underscores the role of genetic and environmental factors in KC development, highlighting the importance of near‐work and light hypersensitivity. Early screening and targeted interventions are recommended, especially for individuals with a family history or related atopic conditions.

## 1 Introduction

Keratoconus (KC) is a bilateral, progressive corneal disorder characterized by thinning and protrusion of the cornea, leading to visual disturbances [1]. It is the most prevalent form of keratectasia, predominantly affecting adolescents. The condition results in refractive errors and corneal opacity, both of which can impair vision and significantly impact quality of life. KC may progress acutely or remain stable, with children or young adults being at higher risk for faster progression [2]. Typically, the disease begins during adolescence and continues to progress until the third or fourth decade of life, after which its progression may stabilize [3]. Children with KC often experience vision distortion and blurring due to irregular astigmatism, myopia, and corneal scarring [4]. If not diagnosed and treated promptly, KC can result in significant vision loss and even blindness, which can affect education, social interactions, and future employment prospects in children [4].

The development and progression of KC have been linked to various factors, including genetic, environmental, systemic, and ocular conditions [3, 5, 6]. Reported systemic conditions include Down syndrome, Ehlers–Danlos syndrome, and asthma [3, 6]. Environmental risk factors include atopy, corneal trauma (such as eye rubbing), ultraviolet (UV) radiation, and sun exposure [5, 6]. Ethnicity has also been identified as a risk factor, with individuals of Indian descent and those from the Middle East being at higher risk due to genetic predisposition and consanguinity, particularly within Muslim communities [7].

Although several factors have been associated with KC, it is important to note that a clear causal relationship has not been established for all of them [3, 4, 5, 6]. Key risk factors proposed in the literature include atopy or allergic conditions, frequent eye rubbing, UV exposure, refractive errors, a family history of KC, the use of rigid gas‐permeable contact lenses, and parental consanguinity. While many studies have examined the prevalence and risk factors of KC globally [3, 4, 5, 8, 9, 10], there is a paucity of studies on KC risk factors in T&T.

The population of T&T may be particularly vulnerable to genetic, sight‐threatening conditions like KC [11] due to its warm climate, dusty environment, and ethnically diverse population, which includes a mix of Black, White, and East Indian communities [8]. Despite a high presentation of KC among adolescents in local ophthalmic services, there is a lack of published studies on KC risk factors within the Caribbean, particularly in T&T. Furthermore, preadmission vision screening before high school is not mandatory, and there are no national school eye screening programs. As a result, children with undiagnosed vision problems related to KC may perform poorly academically, leading to further deterioration in their quality of life. Therefore, this study aimed to assess the risk factors for KC among high school students in T &T is essential. The findings will be valuable in developing targeted strategies aimed at reducing the prevalence and burden of KC in children, ultimately improving their quality of life.

## 2 Methods

### 2.1 Study Design

The study was an institution‐based cross‐sectional study of high school children in T&T to determine risk factors of KC conducted from September 2022 to December 2023. Reporting of this study followed Strengthening the Reporting of Observational Studies in Epidemiology guidelines.

### 2.2 Study Setting and Setting

The study was conducted in T&T, a twin‐island nation situated in the Caribbean, known for its diverse population and rich cultural heritage with a population of ~1.3 million [12], it has a relatively young demographic, with a significant proportion of its population under the age of 18 [13].

### 2.3 Sample Size

The sample size was determined using Raosoft [14], a software used for sample size calculation and the minimum sample size per district was determined as 377 high school students and a total of 2262 (377 × 6) participants nationwide.

### 2.4 Sampling Technique

A multistage stratification cluster sampling technique was used to select participants for the study population. A multistage stratified cluster sampling technique was used to select participants in the study population. Stage 1: Six districts were randomly chosen from the eight districts in T&T (Caroni, Northeastern, Port of Spain, Southeastern, St. George East, St. Patrick, Tobago, and Victoria). Stage 2: Schools in the selected districts were stratified into three categories: full government‐sponsored, partially government‐sponsored, and private schools, comprising a total of 187 secondary schools. Stage 3: Five schools from each selected district were randomly chosen to represent the three school categories in T&T. All students within these selected schools were included in the study population. A cross‐sectional sample of students aged 11–20 was obtained through random cluster sampling. Clusters were defined by the different grade levels (1–5) within each school, resulting in 935 clusters (5 clusters per school across 187 schools). A total of 30 schools (5 schools from each of the 6 districts) were selected, generating 150 clusters (30 schools × 5 grades). Within each cluster, 15 students were initially selected, resulting in 2262 participants. To account for absentees, the sample size per cluster was adjusted to 20, leading to a total of 3000 students across the 30 schools. Stage 4: Systematic random sampling was used to select study participants within each school. Student lists for each grade were obtained from the school registrar or classroom teacher. A sampling fraction was calculated for each school using the formula *N*/*n* where *N* is the total number of students in the class, and *n* is the proportionate allocation. Students were then selected based on this calculated fraction.

### 2.5 Inclusion and Exclusion Criteria

Students aged 11–20 years whose parent or legal guardian provided informed consent to participate in the study were included. Additionally, students who gave signed assent to participate and were residents of T&T, as verified by the class register and classroom teacher, were eligible for inclusion. Students who were unavailable during the study period and those with a history of corneal pathology, traumatic corneal scars, or corneal keratoplasty unrelated to KC were excluded from the study.

### 2.6 Data Collection Tool

The study utilized the Keratoconus Risk Investigative (KRIS) questionnaire for data collection. This questionnaire, which had been used in similar studies conducted in Cameroon [5] and Zimbabwe [15], was modified for the current study. A pilot study, involving 20 students who were not part of the final sample, was conducted to evaluate the validity and reliability of the questionnaire. The questionnaire has questions aimed to identify and assess the risk factors associated with KC among high school children in Trinidad including demographic information, ocular history, family history, general health history, and environmental considerations.

### 2.7 Data Collection Procedure

After obtaining ethical approval from the University of the West Indies Campus Research Ethics Committee, permission was sought from the Ministry of Education to conduct the research in high schools. Following approval, selected schools were visited to request permission from the school principals for student participation in the study. Once approval was granted, arrangements were made for a meeting with the students. During this meeting, the purpose of the study was explained to both the students and their class supervisors. Information documents and consent forms were distributed to the students for their parents or legal guardians to review and sign.

### 2.8 Criteria for Identification of Keratoconus (KC) Risk Factors

Eye rubbing was defined as self‐reported, forceful rubbing of the eyes with the knuckles, with an intensity rating of at least 3 on a 5‐point scale. Parental consanguinity referred to a blood relationship between parents, including first‐ or second‐cousin relationships or similarly close familial ties. Significant sunlight exposure was defined as at least 8 h/week of cumulative exposure to natural sunlight. Near work was characterized by prolonged activities requiring close visual focus, such as reading, smartphone use, or computer work, totaling more than 8 h daily. A positive family history was recorded if a relative had a confirmed KC diagnosis. Atopy was identified based on self‐reported allergic conditions, including asthma, eczema, hay fever, food allergies, pollen, dust, animal fur, or vernal keratoconjunctivitis.

Five key risk factors were identified as well‐established indicators for KC: frequent eye rubbing due to itching, prolonged sun exposure, family history of KC, consanguineous parentage, and a history of atopic diseases. Although additional potential risk factors were evaluated, only these five demonstrated a reliable association with KC. Individuals presenting with at least three risk factors, including at least one from the established group, were referred for comprehensive clinical evaluation. This evaluation included retinoscopy, autorefraction, slit‐lamp biomicroscopy, corneal topography, and optical coherence tomography (OCT). The diagnosis of KC was based on a combination of clinical indicators, including the presence of a scissor reflex, irregular astigmatism identified during retinoscopy, abnormalities detected by topography and OCT, and slit‐lamp findings such as Vogt's striae, Fleischer rings, or Munson's sign.

### 2.9 Ethical Approval

The study adhered to the ethical principles outlined in the Declaration of Helsinki for research involving human subjects. Ethical approval for the study was obtained from the University of the West Indies Research and Ethics Committee (CREC‐SA.0002/08/2019). Permission was also granted by the Ministry of Education and the principals of the selected schools. Prior to data collection, the purpose of the study and the reasons for participation were clearly explained to the participants. Informed consent was obtained from both the participants and their parents.

### 2.10 Data Analysis

The sample characteristics were presented using frequencies and percentages. To assess the differences or associations of KC, odds ratios (ORs) were calculated using *χ* ^2^ test. Binary logistic regression was subsequently performed to investigate these associations further. Adjusted odds ratios (AORs) and their 95% confidence intervals (CIs) were derived from the adjusted multivariable logistic regression model to quantify the relationships. Statistical significance was assessed at a 5% significance level. All statistical analyses were performed using Statistical Package for the Social Sciences (IBM Corp, SPSS Statistics for Windows Version 27.0, Armonk, NY, USA).

## 3 Results

### 3.1 Profile of Study Participants

Of the 3000 students enumerated for the study, 2084 students (69.5%) with a mean age of 14.02 years (SD = 1.60, range 11–20) participated in the study. Almost 60% (58.9%, *n* = 1228) of them identified as female and were of East Indian descent (48.4%, *n* = 1009). The majority were Christians (60.0%, *n* = 1250) and resided in urban areas (52.0%, *n* = 1083) (Table 1).

**Table 1:** Profile of study participants.

Variables	Subgroup	Frequency	Percentage frequency
Nationality	Citizen	2075	99.6
	Noncitizen	9	0.4
Gender	Female	1228	58.9
	Male	856	41.1
Age (mean (SD), range)		14.02 (1.60), 11–20	
Residence	Rural	370	17.8
	Semi‐urban	631	30.3
	Urban	1083	52.0
Ethnicity	African	481	23.1
	East Indian	1009	48.4
	Mixed	494	23.7
	Others (Syrians, Chinese, and Caucasians)	100	4.8
Religion	Christianity	1250	60.0
	Hinduism	544	26.1
	Islam	190	9.1
	Others (Atheists, Orisha, or traditional African religion)	100	4.8
Form	One	277	13.3
	Two	558	26.8
	Three	539	25.9
	Four	296	14.2
	Five	273	13.1
	Six	141	6.8
Has refractive error	Yes	566	27.2
	No	1518	72.8
Total		2084	100.0

### 3.2 Distribution of Risk Factors Among All Participants

The most prevalent risk factor overall (with and without KC) was near task (≥ 8 h/day) (85.9%) followed by experience of hypersensitivity to light (41.7%) and blurry vision (33.3%). In terms of established risk factors, sunlight exposure (25%) was the most prevalent followed by itchy eye or eye rubbing (14.6%) and consanguinity (7.5%) (Table 2).

**Table 2:** Distribution of risk factors among all participants.

Variables	Subheadings	Frequency	Percentage frequency
Has anyone in your family been diagnosed with keratoconus^a^	Yes	103	4.9
	No	1981	95.1
If yes, who? Respondents (*n* = 74)	Aunty	7	0.3
	Cousin	19	0.9
	Father	9	0.4
	Grandmother	6	0.3
	Mother	9	0.4
	Sibling	19	0.9
	Uncle	5	0.2
	No response	2010	96.4
Are your parents blood relatives (consanguinity)^a^	Yes	157	7.5
	No	1927	92.5
How many hours do you spend outside in the sunlight^a^	< 8	1419	68.1
	8–24	520	25.0
	More than 24	145	7.0
Do you have any allergic or atopic diseases^a^	Yes	128	6.1
	No	1956	93.9
Do you experience itchy eyes or eye rubbing^a^	Yes	304	14.6
	No	1780	85.4
How many hours do you spend doing near tasks per day	< 8	293	14.1
	8 or more	1791	85.9
Do you wear/have you worn rigid contact lenses	Yes	67	3.2
	No	2017	96.8
Have you had LASIK eye surgery previously	Yes	48	2.3
	No	2036	97.7
Do you wear spectacles	Yes	556	26.7
	No	1528	73.3
Do you experience glare sensitivity	Yes	503	24.1
	No	1581	75.9
Do you experience blurry vision	Yes	695	33.3
	No	1389	66.7
Do you experience hyperlight sensitivity	Yes	868	41.7
	No	1216	58.3

a Established risk factor.

### 3.3 Demographic Distribution of Participants With Established Risk Factors

Out of a total of 2084 participants (with and without KC) screened, about 674 had at least one established risk factor giving a prevalence of 32.3%. Of the 674 participants with established risk factors, 60 had KC confirmed through further clinical assessment. The majority of those with the established risk factors were of East Indian descent (*n* = 340), females (*n* = 369), Christians (*n* = 365), reside in urban areas (*n* = 349), and in form three (*n* = 180). Established risk factors were found to be associated with gender (*χ* ^2^ = 7.18, df = 1, *p* = 0.007), religion (*χ* ^2^ = 17.01, df = 3, *p* = 0.001), ethnicity (*χ* ^2^ = 9.04, df = 3, *p* = 0.029), and form (*χ* ^2^ = 19.30, df = 5, *p* = 0.002). Females were 1.29 times (95% CI: 1.07–1.55) more likely to have at least one risk factor than males (Table 3).

**Table 3:** Demographic distribution of those with the established risk factors.

Variables	Subgroup	Having at least one established risk factor		Total (%)	*p*
		Yes	No		
Gender	Female	369 (17.7)	859 (41.2)	1228 (58.9)	0.007*
	Male	305 (14.6)	551 (26.4)	856 (41.1)	
Residence	Rural	119 (5.7)	251 (12.0)	370 (17.8)	0.981
	Semi‐urban	206 (9.9)	425 (20.4)	631 (30.3)	
	Urban	349 (16.7)	734 (35.2)	1083 (52.0)	
Ethnicity	African	147 (7.1)	334 (16.0)	481 (23.1)	0.029*
	East Indian	340 (16.3)	669 (32.1)	1009 (48.4)	
	Mixed	144 (6.9)	350 (16.8)	494 (23.7)	
	Others	43 (2.1)	57 (2.7)	100 (4.8)	
Religion	Christianity	365 (17.5)	885 (42.5)	1250 (60.0)	0.001*
	Hinduism	210 (10.1)	334 (16.0)	544 (26.1)	
	Islam	69 (3.3)	121 (5.8)	190 (9.1)	
	Others	30 (1.4)	70 (1.4)	100 (4.8)	
Form	One	97 (4.7)	180 (8.6)	277 (13.3)	0.002*
	Two	147 (7.1)	411 (19.7)	558 (26.8)	
	Three	180 (8.6)	359 (17.2)	539 (25.9)	
	Four	96 (4.6)	200 (9.6)	296 (14.2)	
	Five	92 (4.4)	181 (8.7)	273 (13.1)	
	Six	62 (3.0)	79 (3.8)	141 (6.8)	
Total		674 (32.3)	1410 (67.7)	2084 (100.0)	

*Note:* *Indicates significant association.

### 3.4 Comparison of Risk Factors Between Those With and Without KC

Sunlight exposure (*n* = 21) and family history (*n* = 9) were the most prevalent established risk factors among those with KC while prolonged near activity (*n* = 46) and hypersensitivity to light (19) were the most nonestablished risk factors among participants with KC (Table 4).

**Table 4:** Comparison of risk factors between those with and without KC.

Variables	Subheadings	Do you have KC		Total (%)
		Yes	No	
Has anyone in your family been diagnosed with keratoconus	Yes	9 (0.4)	94 (4.5)	103 (4.9)
	No	51 (2.4)	1930 (92.6)	1980 (95.1)
If yes, who?	None	54 (2.6)	1956 (93.9)	2010 (96.4)
	Aunty	0 (0.0)	7 (0.3)	7 (0.3)
	Cousin	2 (0.1)	17 (0.8)	19 (0.9)
	Father	2 (0.1)	7 (0.3)	9 (0.4)
	Grandmother	0 (0.0)	6 (0.3)	6 (0.3)
	Mother	1 (0.0)	8 (0.4)	9 (0.4)
	Sibling	1 (0.0)	18 (0.9)	19 (0.9)
	Uncle	0 (0.0)	5 (0.2)	5 (0.2)
Are your parents blood relatives (consanguinity)	Yes	6 (0.3)	151 (7.2)	157 (7.5)
	No	54 (2.6)	1873 (89.9)	1927 (92.5)
How many hours do you spend outside in the sunlight	< 8	34 (1.6)	1385 (66.5)	1419 (68.1)
	8–24	21 (1.0)	499 (23.9)	520 (25.0)
	More than 24	5 (0.2)	140 (6.7)	145 (7.0)
Do you have any allergic or atopic diseases	Yes	8 (0.4)	120 (5.8)	128 (6.1)
	No	52 (2.5)	1904 (91.4)	1956 (93.9)
Do you experience itchy eyes or eye rubbing	Yes	8 (0.4)	296 (14.2)	304 (14.6)
	No	52 (2.5)	1728 (82.9)	1780 (85.4)
How many hours do you spend doing near tasks	< 8	14 (0.7)	279 (13.4)	293 (14.1)
	8 or more	46 (2.2)	1745 (83.7)	1791(85.9)
Do you wear/have you worn rigid contact lenses	Yes	0 (0.0)	67 (3.2)	67 (3.2)
	No	60 (2.9)	1957 (93.9)	2017 (96.8)
Have you had LASIK eye surgery previously	Yes	0 (0.0)	48 (2.3)	48 (2.3)
	No	60 (2.9)	1976 (94.8)	2036 (97.7)
Do you wear spectacles	Yes	15 (0.7)	541 (26.0)	556 (26.7)
	No	45 (2.2)	1483 (71.2)	1528 (73.3 s)
Do you experience glare sensitivity	Yes	19 (0.9)	484 (23.2)	503 (24.1)
	No	41 (2.0)	1540 (73.9)	1581 (75.9)
Do you experience blurry vision	Yes	14 (0.7)	681 (32.7)	695 (33.3)
	No	46 (2.2)	1343 (64.4)	1389 (66.7)
Do you experience hyperlight sensitivity	Yes	37 (1.8)	831 (39.9)	868 (41.7)
	No	23 (1.1)	1193 (57.2)	1216 (58.3)

### 3.5 Association of the Risk Factors of KC With the KC Diagnosis

Using Pearson's *χ* ^2^ test, KC was significantly associated with family history (*χ* ^2^ = 13.30, df = 1, *p* < 0.001), allergic diseases (*χ* ^2^ = 5.54, df = 1, *p* = 0.019), and light sensitivity (*χ* ^2^ = 10.18, df = 1, *p* = 0.001). Individuals with a family history of KC were more likely to have KC (0.4% vs. 4.5%). No significant association was found between KC and other risk factors (established and nonestablished) (all *p* > 0.05).

An adjusted logistic regression analysis revealed that individuals with a family history of KC were 2.56 times more likely to have KC (95% CI: 1.11–5.93, *p* = 0.028), while those experiencing light sensitivity had nearly double (2.10) the odds of having KC (95% CI: 1.23–3.59, *p* = 0.007). However, the association between atopic diseases/allergies and KC was not significant (AOR = 1.540, 95% CI: 0.64–3.68, *p* = 0.332) (Table 5).

**Table 5:** Association of the risk factors of KC with KC diagnosis.

Variables	Subheadings	Do you have KC Frequency		Total (%)	*p*	OR (CI)
		Yes	No			
Has anyone in your family been diagnosed with keratoconus	Yes	9 (0.4)	94 (4.5)	103 (4.9)	< 0.001*	3.62 (1.73–7.58)
	No	51 (2.4)	1930 (92.6)	1980 (95.1)		
If yes, who?	None	54 (2.6)	1956 (93.9)	2010 (96.4)		
	Aunty	0 (0.0)	7 (0.3)	7 (0.3)		
	Cousin	2 (0.1)	17 (0.8)	19 (0.9)		
	Father	2 (0.1)	7 (0.3)	9 (0.4)		
	Grandmother	0 (0.0)	6 (0.3)	6 (0.3)		
	Mother	1 (0.0)	8 (0.4)	9 (0.4)		
	Sibling	1 (0.0)	18 (0.9)	19 (0.9)		
	Uncle	0 (0.0)	5 (0.2)	5 (0.2)		
Are your parents blood relatives (consanguinity)	Yes	6 (0.3)	151 (7.2)	157 (7.5)	0.463	
	No	54 (2.6)	1873 (89.9)	1927 (92.5)		
How many hours do you spend outside in the sunlight	< 8	34 (1.6)	1385 (66.5)	1419 (68.1)	0.146	
	8–24	21 (1.0)	499 (23.9)	520 (25.0)		
	More than 24	5 (0.2)	140 (6.7)	145 (7.0)		
Do you have any allergic or atopic diseases	Yes	8 (0.4)	120 (5.8)	128 (6.1)	0.019*	2.44 (1.13–5.26)
	No	52 (2.5)	1904 (91.4)	1956 (93.9)		
Do you experience itchy eyes or eye rubbing	Yes	8 (0.4)	296 (14.2)	304 (14.6)	0.780	
	No	52 (2.5)	1728 (82.9)	1780 (85.4)		
How many hours do you spend doing near tasks	< 8	14 (0.7)	279 (13.4)	293 (14.1)	0.106	
	8 or more	46 (2.2)	1745 (83.7)	1791(85.9)		
Do you wear/have you worn rigid contact lenses	Yes	0 (0.0)	67 (3.2)	67 (3.2)	0.152	
	No	60 (2.9)	1957 (93.9)	2017 (96.8)		
Have you had LASIK eye surgery previously	Yes	0 (0.0)	48 (2.3)	48 (2.3)	0.227	
	No	60 (2.9)	1976 (94.8)	2036 (97.7)		
Do you wear spectacles	Yes	15 (0.7)	541 (26.0)	556 (26.7)	0.765	
	No	45 (2.2)	1483 (71.2)	1528 (73.3)		
Do you experience glare sensitivity	Yes	19 (0.9)	484 (23.2)	503 (24.1)	0.167	
	No	41 (2.0)	1540 (73.9)	1581 (75.9)		
Do you experience blurry vision	Yes	14 (0.7)	681 (32.7)	695 (33.3)	0.095	
	No	46 (2.2)	1343 (64.4)	1389 (66.7)		
Do you experience hyperlight sensitivity	Yes	37 (1.8)	831 (39.9)	868 (41.7)	0.001*	2.31 (1.36–3.92)
	No	23 (1.1)	1193 (57.2)	1216 (58.3)		

*Note:* *Indicates significant association.

A Pearson's *χ* ^2^ test revealed significant associations across various demographic and KC risk factors. Gender was significantly associated with sunlight exposure (*χ* ^2^ = 4.74, df = 1, *p* = 0.029), ocular allergy (*χ* ^2^ = 4.14, df = 1, *p* = 0.042), wearing glasses (*χ* ^2^ = 34.54, df = 1, *p* < 0.001), and glare sensitivity (*χ* ^2^ = 5.53, df = 1, *p* = 0.019). Area of residence was significantly associated with sunlight exposure (*χ* ^2^ = 16.27, df = 2, *p* < 0.001), wearing glasses (*χ* ^2^ = 20.14, df = 2, *p* < 0.001), and visual symptoms like glare sensitivity (*χ* ^2^ = 141.42, df = 2, *p* < 0.001), blurry vision (*χ* ^2^ = 116.87, df = 2, *p* < 0.001), and light sensitivity (*χ* ^2^ = 167.80, df = 2, *p* < 0.001). Religion was associated with multiple visual symptoms such as wearing spectacles (*χ* ^2^ = 67.47, df = 3, *p* < 0.001), glare sensitivity (*χ* ^2^ = 148.70, df = 3, *p* < 0.001), blurry vision (*χ* ^2^ = 60.84, df = 3, *p* < 0.001), and light sensitivity (*χ* ^2^ = 114.81, df = 3, *p* < 0.001). Ethnicity showed significant relationships with wearing glasses (*χ* ^2^ = 77.95, df = 3, *p* < 0.001), and visual symptoms (glare sensitivity [*χ* ^2^ = 54.62, df = 3, *p* < 0.001], blurry vision [*χ* ^2^ = 68.36, df = 3, *p* < 0.001], light sensitivity [*χ* ^2^ = 27.12, df = 3, *p* < 0.001]), as well as rigid contact lens use (*χ* ^2^ = 16.49, df = 3, *p* = 0.001). Additionally, the form of the students was significantly associated with risk of sunlight exposure (*χ* ^2^ = 15.38, df = 5, *p* = 0.009), atopic diseases (*χ* ^2^ = 14.04, df = 5, *p* = 0.015), and multiple visual symptoms (glare sensitivity [*χ* ^2^ = 47.34, df = 5, *p* < 0.001], blurry vision [*χ* ^2^ = 16.81, df = 5, *p* = 0.005], light sensitivity [*χ* ^2^ = 57.85, df = 5, *p* < 0.001]).

## 4 Discussion

This study provides the first assessment of KC risk factors among high school students in T&T. Approximately 32.3% were identified as at risk for KC, with 2.9% diagnosed with the condition. Among all participants, both with and without KC, the most observed primary risk factors were sunlight exposure (established risk factor) and near‐task activities (nonestablished risk factor). Additionally, gender, religion, ethnicity, and academic year were found to influence the presence of at least one risk factor.

Females were more likely than males to have at least one risk factor. Other significant associations included a family history of KC, allergic conditions, and light hypersensitivity, with individuals having a family history and experiencing hypersensitivity to light being double at risk of having the condition.

The prevalence of individuals at risk for KC in this study is consistent with findings from studies in Cameroon (34.46%) and India (36%), suggesting a relatively high at‐risk population in T&T [5, 16]. However, the authors would like to note that the diagnostic criteria for KC may differ between studies, and this is a weakness. The ethnic composition and climatic conditions in T&T may contribute to this elevated risk. The prevalence of KC in the current study aligns with findings from studies in Iran [17], the UAE [7], and Jerusalem [18] but lower than reports from India (3.6%–4.8%) [16, 19] and Saudi Arabia (3.6%–4.8%) [20]. Variations in the prevalence could be due to differences in diagnostic criteria, genetic and environmental factors. Additionally, ethnicity has long been recognized as an important factor in KC development, with studies showing higher susceptibility in certain ethnic groups, including individuals of Middle Eastern, South Asian, and East Asian descent [5, 8, 18].

Sunlight exposure was the most reported established risk factor, likely due to the intense UV rays in T&T's hot climate. Prolonged UV exposure can contribute to oxidative damage in the cornea, which, in conjunction with reduced enzymatic protection, may increase KC susceptibility [19]. This finding aligns with studies from regions with similar climates, such as the Middle East, Israel, India, Cameroon, and Saudi Arabia [5, 8, 9, 16, 21, 22]. This highlights the importance of considering sunlight exposure in KC screening in children as well as importance of wearing UV protective device to reduce the consequences of UV exposure including KC.

Eye rubbing and atopy, while not the most common risk factors in this study, were frequently reported and are known to contribute to KC progression in other studies [5, 8, 21, 23, 24, 25, 26]. Eye rubbing is often associated with allergic reactions, which can cause corneal thinning and exacerbate KC. Moreover, individuals with atopic conditions were double more likely to develop KC. This supports findings from other studies [8, 27] suggesting that allergic diseases, like asthma and eczema, may predispose individuals to KC through immune system dysfunction. While the precise mechanisms remain unclear, chronic inflammation and immune system dysfunction characteristic of atopic diseases may play a role in weakening the corneal structure, making it more susceptible to ectatic changes [28, 29]. This highlights the importance of considering eye rubbing during KC screening in children. Contrary to our study findings, studies in Iran [8], China [30], and Korea [31] did not identify atopy as a significant risk factor of KC. Differences in the definition of atopy used in different studies could be the reason for the variations. The need to establish a uniform definition of atopy cannot be overemphasized to understand the role it plays in the development and progression of KC. To enhance comparability with existing literature, future studies should adopt a standardized definition of atopy based on clinical criteria or validated questionnaires, incorporating a more comprehensive list of allergic conditions associated with KC.

Family history remains one of the most significant risk factors for KC globally [5, 8, 18, 32, 33]. In the present study, participants with a family history of the condition were twice as likely to develop KC in the current study, reinforcing its genetic basis. The role of consanguinity, however, was less influential in this study compared to studies in regions with a higher prevalence of genetic diseases, such as in the Middle East [32, 34, 35], likely due to the low consanguinity rate in the current study compared to that of Saudi Arabia. The present study also recorded frequent reports of near‐task activities and light hypersensitivity as nonestablished risk factors. Though these factors have not been widely studied in relation to KC, individuals reporting light sensitivity were 2.10 times more likely to develop KC in the present study. This suggests a potential connection between light exposure and KC progression, warranting further investigation.

The similarity in the mean age of those at risk of KC in the current study with those from India and Cameroon [5, 16, 19] underscores the need for early KC screening in children. In this study, demographic factors, including gender, ethnicity, and school grade, were significantly associated with risk factors, with females more likely to present with established risk factors. This may reflect hormonal influences on corneal thinning during puberty, though more research is needed to confirm these findings. Similar findings were reported in other studies [5, 16, 26, 36]. However, Abuallut et al. [27] reported no significant association with gender in their study in Saudi Arabia, which is contrary to the findings of this study. This difference between the studies could be due to regional or cultural factors that influence the exposure to or the reporting of risk factors, as well as differences in sample populations. The fact that the impact of gender on KC risk remains unclear suggests the need for more studies to better understand the gender‐based differences in KC risk factors.

### 4.1 Strengths, Limitations, and Future Recommendations

The study addresses a significant health concern, as KC can lead to significant visual impairment and reduced quality of life, particularly in adolescents. The study included a large sample of 2084 high school students, providing a robust data set for analysis. The study used a multistage stratified cluster sampling technique, which enhances the representativeness of the sample. The use of a modified KRIS questionnaire, previously used in similar studies, ensures a degree of standardization.

However, there are some limitations to consider. First, the cross‐sectional design of this study limits our ability to establish causal relationships between risk factors and KC. Longitudinal studies that track changes in visual behaviors and environmental exposures over time would provide more robust evidence regarding the temporal sequence of risk factor exposure and the development of KC. This would help clarify whether factors such as near‐vision activities and photophobia are causal or merely correlational in the development of KC. Second, while our sample consisted of high school students, it may not fully represent the broader population, particularly since the findings may not be generalizable to other age groups or populations with different ethnic and environmental backgrounds. Population‐based studies with broader age ranges, varied geographical settings, and diverse ethnic groups are needed to determine whether the risk factors identified in this study are consistent across different contexts. Such studies would allow for the assessment of interactions between genetic, environmental, and behavioral factors, ultimately contributing to more effective strategies for the prevention, early detection, and management of KC.

Third, the use of self‐report in atopy and the intensity scale of eye rubbing is subjective and prone to recall bias, which may have potentially affected the accuracy of the results. Furthermore, the self‐reported definition and measurement of allergies, while covering common conditions, did not encompass a broader range of allergic conditions consistently defined across studies, potentially affecting the comprehensiveness and comparability of the results regarding atopy's association with KC. Given the limitations of self‐reported data, particularly for allergies and eye‐rubbing behavior, objective measures should be implemented to improve data accuracy. These could include direct observation, validated scales for frequency and intensity, or advanced methods such as time‐lapse photography to quantify eye‐rubbing behavior more precisely. For allergies, future studies should adopt a standardized definition of atopy based on clinical criteria or validated questionnaires, incorporating a more comprehensive list of allergic conditions associated with KC. Fourth, this study did not assess the particular near activities that were associated with an increased risk for KC (e.g., computer use, reading, screen time on electronic devices), thereby limiting the practicality and specificity of the findings regarding these nonestablished risk factors. To reduce the impact of this, future studies should provide greater specificity regarding near‐vision tasks associated with increased KC risk, such as prolonged computer use, reading, or screen time on electronic devices, as the current evidence lacks detailed categorization. Investigating the types, duration, and intensity of these activities will enhance the practical relevance of findings for public health interventions. Further considerations for future studies would include exploration of photophobia (light hypersensitivity) and its possible association with KC and prioritization of genetic research to explore hereditary mechanisms underlying KC, particularly in genetically diverse populations such as those in T&T. Also, it would be valuable to investigate the influence of systemic conditions (e.g., diabetes), sleep positions, and other lifestyle factors on the onset and progression of KC.

### 4.2 Implications of the Findings

The findings of this study have several important implications for the understanding of KC. First, the identification of both established (family history and sunlight exposure) and non‐established (near‐task activities and light hypersensitivity) risk factors offers valuable insights for clinicians and researchers working to understand the multifactorial nature of KC. These findings suggest that both genetic predisposition and environmental factors, including lifestyle choices, may interact in the development of the condition.

Moreover, the identification of near‐task activities as a potential risk factor for KC has practical implications for public health, especially in regions where high school students engage in extended periods of reading and screen time. Public health initiatives and educational programs promoting visual ergonomics and regular breaks from near‐vision tasks could help reduce the potential risk for KC development, particularly in younger populations.

## 5 Conclusion

This study provides valuable insights into the risk factors associated with KC in T&T and largely aligns with existing research, while also offering novel insights into the risk factors associated with KC among high school students in T&T. The results emphasize the significant role of both genetic and environmental factors in the development of KC, with particular attention to near‐task activities and light hypersensitivity as potential contributors. These findings highlight the need for early screening and targeted interventions, particularly for individuals with a family history of KC or related atopic conditions. Future research should focus on longitudinal environmental and genetic studies to further investigate these risk factors, enhancing our understanding of the etiology of KC and informing the development of effective prevention strategies.

## Author Contributions

Conception and design: Ngozika Esther Ezinne. Material preparation and data collection: Ngozika Esther Ezinne, Shinead Phagoo, and Ameera Roopnarinesingh. Data analysis and visualization: Michael Agyemang Kwarteng. Supervision: Khathutshelo Percy Mashige and Uchechukwu Levi Osuagwu. All authors contributed to writing the original draft, editting, reviews and writing revisions. All authors read and approved the final manuscript. Ngozika Esther Ezinne had full access to all the data in this study and takes complete responsibility for the integrity of the data and the accuracy of the data analysis.

## Funding

The authors received no specific funding for this work.

## Disclosure

The lead author Ngozika Esther Ezinne affirms that this manuscript is an honest, accurate, and transparent account of the study being reported; that no important aspects of the study have been omitted; and that any discrepancies from the study as planned (and, if relevant, registered) have been explained.

## Ethics Statement

The study adhered to the ethical principles outlined in the Declaration of Helsinki for research involving human subjects. Ethical approval for the study was obtained from the University of the West Indies Research and Ethics Committee (CREC‐SA.0002/08/2019).

## Consent

Informed consent was obtained from both the participants and their parents.

## Conflicts of Interest

The authors declare no conflicts of interest.

## Data Availability

The data used in the study are available from the corresponding author upon a reasonable request.
